# Managing symptoms during cancer treatments: evaluating the implementation of evidence-informed remote support protocols

**DOI:** 10.1186/1748-5908-7-110

**Published:** 2012-11-19

**Authors:** Dawn Stacey, Debra Bakker, Barbara Ballantyne, Kimberly Chapman, Joanne Cumminger, Esther Green, Margaret Harrison, Doris Howell, Craig Kuziemsky, Terry MacKenzie, Brenda Sabo, Myriam Skrutkowski, Ann Syme, Angela Whynot

**Affiliations:** 1 School of Nursing, Faculty of Health Sciences, University of Ottawa, Ottawa, ON, Canada; 2 School of Nursing, Laurentian University, Sudbury, ON, Canada; 3 Northeast Cancer Centre, Health Sciences North, Sudbury, ON, Canada; 4 Horizon Health Network, Fredericton, New Brunswick, Canada; 5 Pictou County Health Authority, New Glasgow, NS, Canada; 6 Cancer Care Ontario, Toronto, ON, Canada; 7 School of Nursing, Queen’s University, Kingston, ON, Canada; 8 University Health Network, Princess Margaret Hospital, Toronto, ON, Canada; 9 Telfer School of Management, University of Ottawa, Ottawa, ON, Canada; 10 Sudbury Regional Hospital, Regional Cancer Program, Sudbury, ON, Canada; 11 School of Nursing, Dalhousie University, Halifax, NS, Canada; 12 Cancer Care Mission, Nursing Department, McGill University Health Centre, Montreal General Hospital, Montreal, QC, Canada; 13 Canadian Partnership Against Cancer, Toronto, ON, Canada; 14 Capital Health, Halifax, NS, Canada

**Keywords:** Cancer, Symptom management, Implementation science, Mixed-methods study, Guidelines based

## Abstract

**Background:**

Management of cancer treatment-related symptoms is an important safety issue given that symptoms can become life-threatening and often occur when patients are at home. With funding from the Canadian Partnership Against Cancer, a pan-Canadian steering committee was established with representation from eight provinces to develop symptom protocols using a rigorous methodology (CAN-IMPLEMENT^©^). Each protocol is based on a systematic review of the literature to identify relevant clinical practice guidelines. Protocols were validated by cancer nurses from across Canada. The aim of this study is to build an effective and sustainable approach for implementing evidence-informed protocols for nurses to use when providing remote symptom assessment, triage, and guidance in self-management for patients experiencing symptoms while undergoing cancer treatments.

**Methods:**

A prospective mixed-methods study design will be used. Guided by the Knowledge to Action Framework, the study will involve (a) establishing an advisory knowledge user team in each of three targeted settings; (b) assessing factors influencing nurses’ use of protocols using interviews/focus groups and a standardized survey instrument; (c) adapting protocols for local use, ensuring fidelity of the content; (d) selecting intervention strategies to overcome known barriers and implementing the protocols; (e) conducting think-aloud usability testing; (f) evaluating protocol use and outcomes by conducting an audit of 100 randomly selected charts at each of the three settings; and (g) assessing satisfaction with remote support using symptom protocols and change in nurses’ barriers to use using survey instruments. The primary outcome is sustained use of the protocols, defined as use in 75% of the calls. Descriptive analysis will be conducted for the barriers, use of protocols, and chart audit outcomes. Content analysis will be conducted on interviews/focus groups and usability testing with comparisons across settings.

**Discussion:**

Given the importance of patient safety, patient-centered care, and delivery of quality services, learning how to effectively implement evidence-informed symptom protocols in oncology healthcare services is essential for ensuring safe, consistent, and effective care for individuals with cancer. This study is likely to have a significant contribution to the delivery of remote oncology services, as well as influence symptom management by patients at home.

## Background

Most people receive their cancer treatments on an outpatient basis and manage their side effects of the disease and treatments while at home. Remote support provided by nurses using the telephone and/or email is essential to helping patients safely manage their symptoms at home and triage potentially life-threatening symptoms. Despite the fact that remote services by nurses require use of protocols to minimize risk, the most commonly used protocols are out of date and clinical practice guidelines for symptoms are not developed for remote support.

The overall aim of this study is to build an effective and sustainable approach for implementing new evidence-informed protocols for nurses to use when providing remote symptom assessment, triage, and guidance in self-management for patients experiencing symptoms while undergoing cancer treatments. Specific objectives of this study are to

(a) assess barriers influencing nurses’ use of protocols for remote symptom support;

(b) adapt symptom protocols for local use, design implementation strategies, and implement;

(c) monitor use and evaluate outcomes of using symptom protocols for remote support.

Although remote support is a necessary aspect of cancer health services, the ability to deliver best practices using this approach is not well understood. Systematically implementing symptom protocols for remote support will ultimately ensure that nurses are accessing current evidence-based knowledge to assess, triage, and help patients manage cancer treatment-related symptoms.

### The knowledge tools

A set of 13 symptom protocols developed with funding from the Canadian Partnership Against Cancer comprise the knowledge translation (KT) tools to be implemented in this study [1,2]. The protocols were developed using a systematic process guided by the CAN-IMPLEMENT^©^ methodology [3,4]. First, a pan-Canadian Oncology Symptom Triage and Remote Support (COSTaRS) Steering Committee representing eight provinces and including researchers, an information systems researcher, library scientist, and knowledge users (KUs) was convened. KUs included advanced-practice nurses and nurse leaders. Then, systematic reviews were conducted to identify clinical practice guideline(s) for *symptoms* published since 2002, except for three symptoms (fatigue, anxiety, depression) for which there were new guidelines developed by pan-Canadian panels using rigorous processes [5,6]. Guidelines are syntheses of the best available evidence and are designed to support decision making in practice and health policy [7]. Given that identified clinical practice guidelines were not adequate for remote symptom support, 13 symptom protocols were developed based on the available clinical practice guidelines (median three guidelines per protocol; range = 1 to 7). In total, 43 practice guidelines were identified and their quality was appraised using the AGREE instrument (median rigor score 84%; range = 11% to 87%) [8]. Higher rigor scores indicate higher confidence that potential biases in guideline development were addressed, and recommendations are valid (both internally and externally) and feasible for practice [9].

In developing the 13 symptom protocols, the criteria on the AGREE rigor subscale items (*e.g.*, explicit recommendations, linked to evidence, based on systematic review, reviewed by experts) were met. Each protocol includes relevant questions from the valid and reliable Edmonton Symptom Assessment System (ESAS), a widely used screening instrument for routinely identifying symptoms in cancer patients seen in Canadian programs [10,11]. Importantly, the protocol format was designed to enhance usability in remote support practice and with the potential to integrate into an electronic health record. Finally, plain language was used to facilitate communication between nurses using the protocols and patients/families. Each symptom protocol has five recommendations for the nurse: (a) assess symptom severity, (b) triage patient for symptom management based on highest severity, (c) review medications being used for the symptom, (d) review self-management strategies (presented using motivational interviewing techniques [12]), and (e) summarize and document the plan agreed upon with the patient (see Additional file 1: Figure S1).

One of the key challenges in KT is information integration with users, workflows, and contexts. Usability, defined as the ability of users to carry out a task safely, efficiently, and enjoyably [13], is an essential aspect of KT. Preliminary usability testing of our symptom protocols by cancer nurses revealed that they are easy to read, provide just the right amount of information, use appropriate terms, are likely to fit with clinical work flow, and have excellent self-management strategies. In validating the protocols, cancer experts across Canada identified the need for local adaptation to integrate the protocols with their current approaches for handling remote symptom assessments.

In summary, 13 user-friendly remote symptom protocols based on a synthesis of the best available evidence (KT tools) were developed, validated with KUs, and use plain language to facilitate KT to patients. The next logical step is to implement the symptom protocols into routine remote support practices. This study protocol is focused on their implementation in three different oncology programs.

### The evidence-practice gap justifying the study

Prompt and accurate cancer symptom management is an important safety issue given that symptoms can become life-threatening and often occur when patients are at home. For cancer patients, the telephone is the quickest and main route for access to cancer services [14,15]. Thus, an important supportive service for patients is remote access to health professionals by telephone or email for guidance in self-care and triaging symptoms to the appropriate level of care. According to nursing professional practice guidelines, key elements necessary for quality telephone-based services that minimize the risk of litigation are access to protocols, documentation of calls, quality assurance monitoring, and training [16-18]. Despite widespread availability of clinical practice guidelines for symptom management, they are not being used in clinical practice provided remotely [14,19].

The research team conducted two studies: an Ontario-wide survey of ambulatory cancer telephone nursing services and a survey of Canadian cancer nurses [14,19]. Both studies revealed that there was inconsistent use of symptom protocols (often a reference on the shelf) when nurses provide remote support by telephone. The highest clinical priorities nurses identified as requiring symptom protocols were fatigue, anxiety, pain, depression, nausea, and constipation. These are similar symptoms to those reported by 45,118 patients in ambulatory cancer clinics in Ontario when screened using ESAS [11]. In both of the surveys, the most commonly cited protocols were those from Cancer Care Ontario that were available in English and French since 2004 (but recently withdrawn). Barriers to using protocols included (a) limited access to or awareness of protocols, (b) nurses being neutral about having adequate knowledge to manage symptoms remotely, (c) complexity of patients with multiple symptoms, (d) inconsistencies with physician practices, (e) inadequate time, (f) lack of electronic protocols, and (g) outdated evidence underlying protocols.

Finally, for this proposal and to inform implementation, a systematic review of the literature was conducted to determine what symptoms were reported in studies of cancer patients attending emergency rooms [20]. The most common symptoms on presentation were fever with neutropenia, infection, pain, fever, and shortness of breath (N = 16 studies). For studies reporting these symptom presentations, 60% of emergency visits resulted in hospital admissions (range = 31 to 100; 14 studies) and 17% resulted in death (range = 4 to 67%; 10 studies). These findings confirmed the common symptoms and those requiring prompt assessment, triage, and management.

KUs for this study are defined as nurses who provide remote symptom support and managers and/or advanced practice nurses facilitating the provision of these services. Using an integrated KT approach, KUs involved on the COSTaRS Steering Committee oversaw the development of the 13 symptom protocols and participated in their validation. This proposed study continues to involve these KUs in the design of this research proposal and plans to include them in the interpretation of findings. New KUs from three settings involved in this study were added. Their role is instrumental in the development of new knowledge to determine effective approaches for implementing symptom protocols for remote support. Previous research shows that involving KUs throughout the research process is a strong predictor that evidence-based knowledge will be used [21]. Therefore, the aim is to generate knowledge that can be used to facilitate widespread implementation in other jurisdictions in Canada.

In summary, the transfer of research evidence into clinical practice can be slow and haphazard [22,23] and this is no different for remote support using symptom protocols. Despite availability of clinical practice guidelines and symptom protocols, they are not being widely used in practice, there are barriers to their use, and studies are required to determine best practices for implementing them in the processes of routine remote support. To address this shortfall, the next logical step is to collaborate with the KUs in locally adapting the protocols and evaluating practical approaches for implementing them in various contexts that provide remote support.

### Study fit with the funding opportunity

This study is aligned directly with the objectives outlined in the Canadian Institutes of Health Research’s Knowledge to Action funding opportunity announcement by focusing on (a) evaluating interventions designed to increase uptake and application of evidence-based symptom protocols to guide nurses providing remote symptom support; (b) strengthening partnerships between researchers and cancer nurses (KUs) across Canada; (c) enhancing our understanding of knowledge application by using a comparative case study with mixed methods (*e.g.*, survey, focus groups, usability testing, chart audit) to understand influential factors and effective approaches for implementing and sustaining use of KT tools (*e.g.*, symptom protocols) in routine practice; and (d) determining the impact of the study on patient, nurse, and healthcare-system outcomes. Given the importance of patient safety, we believe this study will strengthen the use of evidence in remote symptom management, increase our understanding of how these KT tools can be used in everyday remote clinical practice, and ultimately improve and standardize the way patients are assessed, triaged, and guided to manage cancer treatment-related symptoms.

Therefore, we plan to bridge the gap between evidence (*e.g.*, the clinical practice guidelines) and the practice of remote support by providing KT tools (*e.g.*, symptom protocols) [24].

## Methods

### Study design

A comparative case study will be guided by the Knowledge to Action Framework [22,25] to evaluate the process of implementing evidence-based symptom protocols, determine how they are used, and measure outcomes. This type of in-depth empirical inquiry was chosen to investigate a bounded system (*e.g.*, implementation of protocols in one cancer program) using multiple sources of data within its real-life context and subsequently make comparisons across cases [26]. At the core of the Knowledge to Action framework is Knowledge Creation, a funnel leading to more tailored knowledge that is based on individual studies, then synthesized with systematic reviews or clinical practice guidelines, and finally transferred into KT tools. For this proposal, KT tools are the 13 symptom protocols. The methods for this proposal hail directly from the outer Action Cycle of the framework that is activated by the recognition of a problem by KUs (*e.g.*, patients with cancer experience potentially life-threatening symptoms) and is followed by identification, review, and selection of knowledge relevant to the problem (*e.g.*, symptom protocols). The knowledge is then adapted to the local context. Barriers to knowledge use are assessed and interventions introduced to overcome known barriers. In subsequent phases, knowledge use is monitored, outcomes evaluated, and strategies for sustained knowledge use identified.

### Setting

Three cancer programs in Canada will be invited to participate in this study. To be eligible, cancer programs needed to provide remote support for symptom assessment, triage, and management by nurses for patients receiving cancer treatments. To enhance transferability of findings, oncology programs will be purposely selected from different provinces, representing different characteristics (*e.g.*, urban and rural, various models of remote nursing services, paper-based and electronic documentation) and known to have established relationships between KUs and members of the COSTaRS Steering Committee.

### Data collection tools and procedures

An advisory KU team will be established at each of the three settings to guide the implementation of the protocols, handle issues arising from the study, and ensure an integrated KT approach. Use of committees has been shown to enhance uptake of evidence in nursing practice [27]. Based on the Knowledge to Action Framework [22] and consistent with the research objectives, the proposed study procedures include three phases.

### Phase I: Assess barriers influencing nurses’ use of protocols for remote symptom support

Given the need to tailor interventions to known barriers and facilitators likely to influence implementation of evidence by KUs [3,4,21,24,28,29], a principal step in this study is to assess the factors influencing nurses’ uptake of the protocols as part of routine remote symptom support.

Interviews and/or focus groups will be conducted to determine (a) current practice for providing remote symptom support, (b) potential factors likely to influence use of 13 new symptom protocols, (c) local adaptations for the protocols to enhance use, (d) interventions required to implement the protocols and overcome known barriers, and (e) ways to improve access to the protocols. Purposeful sampling will be used at each of the three settings to reach the following levels: nurses (n = 2), managers/supervisors (n = 1–2), advanced practice nurses/educators (n = 1–2), and patients who have received remote support during cancer treatments within three months (n = 5) (N = 30). The research assistant will conduct the interviews/focus groups and make field notes at the end of the sessions. The interviews/focus groups will be audiotaped and transcribed. Participants will complete demographic questions.

### Survey of factors influencing use of protocols

All nurses who provide remote symptom support, as well as managers and advanced practice nurses/educators who support them at each of the three settings, will be asked to complete the survey of factors influencing use of symptom protocols. Participants will receive a mailed package that will include a cover letter with the study purpose, a copy of the symptom protocols, and the survey with a stamped self-addressed envelope. Procedures for administering the survey will be based on the Dillman’s Tailored Design Method [30]. To enhance response, reminders will be sent to nonresponders at two, four, and five weeks for a total of four contacts [30]. The survey questionnaire includes about 50 statements that measure nurses’ (a) attitudes toward the symptom protocols; (b) perceptions of their knowledge, skills, and competence in using protocols when providing remote symptom support; (c) current practice providing remote support; (d) willingness to use them with patients undergoing cancer treatment; and (e) perception of environmental factors influencing their use. Participants will be asked to rate each statement on a five-point scale ranging from 1 (strongly agree) to 5 (strongly disagree) and neutral in the center. Open questions will explore the need for local adaptation of the symptom protocols to improve the fit with current remote support practices and overcome barriers to their use. The survey items have face validity, and problematic items were removed using principal component analysis in a study to assess physicians’ intentions to use a KT tool [31]. The survey has also been used in studies to assess factors influencing nurses’ use of decision aids in a primary care call center [32] and a cancer call center [33]. The survey tool will be finalized, as necessary, based on the interviews/focus groups.

### Phase II: Adapt protocols for local use, design implementation strategy, and implement

Based on findings from phase I and collaborating with the three advisory KU teams, KT tools will be locally adapted (*e.g.*, symptom protocols), and interventions to overcome barriers to implementation at the patient, nurse, and healthcare-system levels will be selected. Collaborating with KUs when making changes and designing strategies to meet outcomes is an effective approach for facilitating sustained knowledge use [34].

### Adapt symptom protocols

The local advisory KU team will discuss and reach agreement on adaptations to the symptom protocols required for their context. This is a critical step in the Action Cycle of the framework [3,4,22]. This step of adapting to the local context is the process by which KUs make decisions about the appropriateness of the specific knowledge for their circumstances and tailor the knowledge to their setting. If adaptations are required, changes will be made to the protocols, ensuring fidelity of the intervention. Fidelity requires implementing all essential components of the interventions as intended [35]. For example, the symptom protocols may change in their format and how they are linked with information systems in practice but the evidence in the protocols will remain consistent with clinical practice guidelines.

### Select and tailor interventions to the settings

The identified barriers and facilitators to implementing the symptom protocols (phase I) and evidence regarding effective interventions for changing practitioners’ practice [7,27,28] will inform this step of the Action Cycle. When interventions are designed to overcome the barriers at the level of the patient, practitioner, and/or healthcare system, they are more likely to be effective [22,28]. Two systematic reviews found that effective interventions to change nurses’ behaviors are educational interventions and use of committees [27,36]. However, these reviews also indicated the need for more research and the need to conduct studies guided by theoretical frameworks. The Cochrane Effective Practice and Organization of Care (EPOC) group has several systematic reviews of health professional and organizational interventions to improve uptake of evidence in practice. Effective interventions to change health professionals’ behaviors include educational meetings (11–22% change), reminders (14.1%), local opinion leaders (10%), audit and feedback (5%), educational outreach (4.9%), and printed educational materials (4.9%) [21,37]. Furthermore, remote support in itself presents special barriers and challenges, including the need for enhanced communication skills to assess symptoms when the patient cannot be observed, more limited access to the physician, and enhanced skills for using information technology applications [38,39].

### Implementing the symptom protocols

Based on feedback from phase I and the team’s previous research [1,14,19], it is anticipated that implementing the symptom protocols will require (a) providing training for nurses in how to use symptom protocols for remote support and how to better support patients reporting multiple symptoms, (b) integrating symptom protocols into the process of care, and (c) initiating monthly case rounds. To inform the training materials, an environmental scan will be conducted of remote support cancer training programs through the COSTaRS Steering Committee members and by contacting cancer programs in Canada known to provide remote support. Methods for the scan will be based on a recent scan of shared-decision-making training programs [40]. Within a week of the original remote support encounter, nurses will be asked to make follow-up contact to re-assess the symptom using the ESAS symptom severity question [10] and assess patient satisfaction with the remote support [41]. Given that a key element influencing sustainability of nurses’ implementation of KT tools in practice include leadership and booster education [34,42], there will be monthly “case rounds.” At these sessions, nurses will be encouraged to present case reports in which the symptom protocols worked well and/or did not and discuss issues influencing implementation of the symptom protocols in practice. Using case reports, best practices for remote symptom support for use in training will be identified. Other interventions will be considered for improving access to the protocols when providing remote support, developing nurses’ skills in remote support (*e.g.*, communication), and electronic applications for protocols. Electronic applications can improve access to protocols, ensure standardized assessments, improve documentation of remote symptom support, coordinate care across different providers, and facilitate monitoring of trends and quality of remote support provided [14].

### Phase III: Monitor use and evaluate outcomes of using protocols for remote support

During and after implementing KT tools, the next phases in the Action Cycle include monitoring knowledge use, evaluating outcomes, and determining sustained knowledge use [22,25]. Protocol use will be monitored and outcomes evaluated by conducting usability testing and a retrospective chart audit and monitoring remaining barriers interfering with use.

### Usability testing

As part of the process to implement the symptom protocols, think-aloud usability testing will be conducted to determine nurses’ use of the protocols, the ease of use, usefulness for decision making, consistency across users, and functionality of their design. Given that 80% of usability problems are detected with four to five subjects [43], this will be conducted with a minimum of five nurses who routinely provide remote symptom support at each participating site (N = 15). Usability testing will involve studying KUs while they carry out representative tasks using the symptom protocols [44]. As well, we will use think-aloud methods, with some participants talking aloud as they are doing the usability testing (concurrent) and others talking aloud after the usability testing (retrospective). Previous studies have found that using concurrent and retrospective think-aloud methods produce similar results but from different ways (*e.g.*, usability issues are more verbalized with retrospective methods and more observed with concurrent methods) [45]. The research assistant will conduct usability testing using a guide with prompts and audiotape the sessions.

### Monitoring use and evaluating outcomes

We will initially monitor use during the “case rounds” sessions and then informally by the advisory KU team. Through these informal approaches, the team will be able to identify issues early and make changes as necessary to facilitate use of protocols. A retrospective chart audit of nursing documentation will be conducted for a sample of calls by patients experiencing cancer treatment-related symptoms. At each of the three settings, 100 patient calls will be selected using a table of random numbers from the complete list of calls that occurred during months three to six after implementing the protocols. Based on previous studies of telephone advice by nurses [14,38,39,46], the chart audit will be conducted using a standardized form that includes (a) characteristics of the remote symptom support provided (*e.g.*, symptom reported, use of a symptom protocol, plan at end of the remote support); (b) characteristics of the patient (*e.g.*, age, type of cancer treatment, sex, length of time since starting current treatment); and (c) outcomes (*e.g.*, disposition, appropriateness of disposition, symptom resolved, health service use [prescription, emergency visit, hospital admission, death]). Findings on the proportion of calls documented, completeness of documentation, and triage appropriateness will be discussed with the advisory KU teams within each setting, and a summary of findings will be communicated to nurses who provide remote symptom support. Audit and feedback has been shown to be an effective strategy for changing health professionals’ behaviors [47].

After six months of implementation, nurses will be asked to complete a survey to determine their satisfaction with the symptom protocols and whether barriers influencing use of protocols were addressed or new barriers surfaced. Survey methods will be those described in phase I with a modified version of the survey.

### Data analysis

Analysis will be conducted to respond to our research objectives. Qualitative analysis of transcripts will include content analysis of the interview/focus group and usability testing transcripts, which will be done independently by the research assistant and a graduate student. Analysis of usability testing transcripts will use an existing framework that has several categories, including understandability and readability of information content, ease of use, and fit with clinical workflow [44]. Analysis using NVivo 9 (QSR International, Inc., Cambridge, MA, USA) will involve (a) reading each transcript and paired field note in its entirety to identify overarching themes, (b) analyzing transcripts line by line to identify themes, and (c) comparing findings between coders. The unit of analysis will be the setting with comparisons across the three settings. Memos of decisions and code manuals with definitions will be maintained for auditing.

### Quantitative analysis of survey data

Barriers and facilitators to using symptom protocols, chart audit data on use of protocols, and demographics of participants will be coded numerically and entered into SPSS^©^ Statistics (IBM, Armonk, NY, USA) software. Descriptive analysis of the survey items will be conducted to inform the research objectives. The main analysis for the primary outcome, use of protocols for symptom calls in patients receiving chemotherapy and/or radiation therapy, will be descriptive and will include a measure of the prevalence of use across all three settings. Demographics of the participants will be assessed using frequency distribution and univariate descriptive statistics. Paired *t*-tests will be used to detect differences between baseline and post-implementation scores for factors influencing symptom protocol use.

### Mixed-methods analysis

Qualitative and quantitative findings will be triangulated using NVivo to inform our research objectives. A case study will be compiled for each of the three settings from all of the qualitative and quantitative data. Summary data displays will be developed to allow comparisons across the three settings.

### Strengths and limitations of the study

One strength of the study is that the techniques that will be used to enhance the credibility and dependability of the qualitative findings [48] include transcribing audiotaped interviews and focus groups, conducting data analysis independently by two team members, and maintaining a clear audit trail. Another strength is the focus on trying to understand how to implement the protocols within different health systems (macro level) and with front-line nurses (micro level). For the surveys at baseline and post-implementation, there is the potential for nonresponse bias and self-report bias. To minimize nonresponse bias, we will provide reminders to nonresponders according to Dillman’s Tailored Design Method [30]. To minimize any potential impact of self-reported bias, we plan to triangulate data across multiple sources. To enhance internal validity and transferability of findings from the retrospective chart audit, charts will be randomly selected using a random numbers table, data will be collected by trained research assistants using a standardized form, and duplicate data entry will be used to minimize errors. To enhance credibility and transferability of the case studies [26], we will triangulate data across the multiple sources (including qualitative and quantitative data), develop rich descriptions of each setting, and ask KUs to provide feedback on the draft cases.

### Engaging knowledge users as team members

In this integrated KT approach, KUs have been and continue to be key members of the research team. We have already demonstrated collaborations with KUs being members of the COSTaRS Steering Committee (KC, MS, EG) and by reaching out to KUs during the preliminary usability testing and validation of the 13 symptom protocols [49]. For this proposal, we have added more KUs to the research team (BB, AW, TM, JC) to ensure representation from each of the three settings. The KUs will participate on the local advisory KU team (together with local researchers, research assistant, principal investigator) and, as part of their role on the advisory KU team, assist with (a) raising awareness about the study in their setting, (b) help facilitate data collection by research assistants (*e.g.*, focus groups, survey, chart audit), (c) discuss and reach agreement with researchers on the local adaptations required for the symptom protocols, (d) advise on the selection of intervention strategies for implementation, (e) lead case rounds, and (f) help analyze and interpret findings. By engaging KUs in the preliminary work of developing and validating the symptom protocols and designing the proposed study, we are ensuring user-friendly KT tools that are relevant to and more likely to be used by nurses providing remote symptom support [21].

### End of grant knowledge translation

Fundamental in this study is an integrated KT approach whereby KUs are collaborating as team members and the study is designed to have findings of use to them. The KUs will function as knowledge brokers [21] within their organizations and extending into their cancer networks (*e.g.*, associated home-based nursing programs). Once all the data from the three phases are collected and analyzed, we will reconvene the COSTaRS Steering Committee to discuss the findings and establish a plan for implementing successful strategies with other programs across Canada. Five researchers are faculty members in schools of nursing where they can disseminate findings. Our team has an established track record of productive meetings in the process of developing and validating the symptom protocols.

Finally, we plan to publish the proposal and results in open access peer-reviewed journals and provide presentations at relevant national and international meetings, including the annual meeting of the Canadian Association of Nurses in Oncology and the International Society of Nurses in Cancer Care (for which many of the researchers and KUs are members). We also plan to disseminate our results through relevant websites (*e.g.*, http://www.ktcanada.ohri.ca).

### Feasibility and timeline

We have a highly productive and experienced team (N = 15) consisting of researchers (n = 7), KUs (n = 7), and a collaborator (n = 1) who are specialized in cancer patient care (n = 13), KT research (n = 5), information systems (n = 1), and health services research (n = 10). Our team has collaborated on a series of studies informing this proposal [1,14,19,20]. An executive committee composed of the principal researchers (DS, DB), principal KUs (EG, AW, BB), and research assistant (MC) will oversee the study. The local advisory KU Team will meet with the principal investigator and research assistant routinely. The full committee will receive monthly updates on study progress.

Our team has the necessary expertise, track record of collaborative studies informing the current proposal, and collaborations with KUs to not only conduct the proposed study but also transfer findings into the nursing practice, education, applied health services research, and health policy. The team members represent six provinces and various research, clinical, and policy-making roles to complement each other’s expertise and provide synergistic potential.

This study will be conducted over two years (Table 1). After obtaining ethics approvals, we plan to stagger the initiation of the study within each of the three settings at one-month intervals to allow time to learn from each setting and make adjustments as the study proceeds. Within each setting, we will conduct the barriers assessment and adapt the protocols prior to usability testing, implement the protocols, and evaluate protocols use and outcomes. At the end of the study, a meeting will be convened with the COSTaRS Steering Committee to discuss findings and plan for broader implementation across Canada.

**Table 1:** Study timeline

**Tasks**	**Month**											
	**2**	**4**	**6**	**8**	**10**	**12**	**14**	**16**	**18**	**20**	**22**	**24**
1. Ethics approval at three settings	X	X										
2. Establish advisory KU teams	X											
3. Focus groups/interviews, analysis			X	X								
4. Survey with reminders, analysis				X	X							
5. Adapt protocols for use					X	X						
6. Environmental scan: training		X										
7. Design intervention toolkit						X						
8. Implement protocols							X					
9. Test usability, analysis							X					
10. Evaluate protocol use and outcomes								X	X	X		
11. Repeat survey with reminders										X	X	
12. End-of-study KT meeting												X

KU = knowledge user; KT = knowledge translation.

### Anticipated outcomes for nursing practice, patients, and health services use

Effective symptom management is an essential component of nursing care that has been shown to decrease symptom severity, improve quality of life, and lower health service use [50,51]. A study of symptoms and treatment burdens associated with cancer treatment found that 28% of patients reported a clinical visit directly related to the symptom experienced [52]. In some cases, these symptoms can be managed through remote support services; however, others may be life-threatening and require immediate care [53]. Given that symptom protocols are evidence-informed and will have been adapted to local contexts, the team anticipates that their use will have a positive impact on enhancing nurses’ practice and competencies for providing remote support, which will lead to improved patient health outcomes with better symptom control. As a result of the impact on nurses and patients, it is expected there will be a positive impact on the health system, with patients receiving more appropriate levels of care (including self-care) for their symptoms.

### Enhanced remote nursing practice

Standards of practice explicitly indicate the need for protocols to guide remote nursing practice, improve accountability to prevent foreseeable or actual harm, and minimize risk of litigation [16-18]. It is anticipated that there will be practice changes with using the symptom protocols and that using them will have a positive impact on nurse outcomes, such as feeling more satisfied with their new approach to providing remote symptom support and more knowledgeable about the current evidence for symptom management. The goal in this study is to have nurses using symptom protocols for 75% of their remote symptom support encounters with patients undergoing chemotherapy and/or radiation therapy.

### Improve symptom management for patients

It is expected that if patients experiencing symptoms at home contact the nurse to be assessed, triaged, and guided in self-management of their symptoms, the patients will feel more confident with managing their symptoms at home and their symptoms (when appropriate) will be controlled or improved. Inherent in the design of the symptom protocols is the expectation that a patient outcome will be improved symptom control. These protocols were based on the current state of knowledge (using clinical practice guidelines) for managing cancer treatment-related symptoms [1]. As discussed earlier, the symptom protocols were developed using principles of motivational interviewing [12]. Previous research has shown that patients exposed to brief motivational interviewing are more likely to make a change [54]. Another important element in the protocols is triaging patients to a higher level of care for severe symptoms requiring more intensive interventions. Therefore, another anticipated patient outcome is that patients are likely to learn when it is important to call and report a symptom, which may result in earlier recognition of impending emergency situations, leading to decreased deaths. For example, fewer patients will be dying from adverse events of cancer treatment that they shouldn’t have died from (*e.g.*, chemotherapy for early-stage cancer often causes low blood counts that can lead to an increased risk of a serious infection that can cause death).

### Improved use of cancer healthcare services

It is anticipated that there will be more appropriate use of healthcare services as a result of nurses using the symptom protocols. More specifically, the expectation is to see fewer in-person visits and emergency room visits, and of those resulting in emergency room visits, they will be determined to be appropriate. A systematic review of telephone advice services in primary care found 50% of calls could be handled by telephone alone, without an in-person or emergency department visit [46,55]. Compared to usual care, there was no difference in deaths when nurses gave the advice.

### Plan to assess study impact on anticipated outcomes

An important element of the Action Cycle is outcome evaluation. Although we identified a number of anticipated outcomes at the level of the patient, the nurse, and the healthcare system, this evaluation plan will focus on a limited set of more immediate outcomes. Earlier, we discussed the evaluation plan for process outcomes (*e.g.*, use of protocols, barriers to nurses using the protocols, change in barriers, usability), including the evaluation to determine effectiveness of the KT interventions. The following focuses on the plan to measure outcomes at the level of the nurse, patient, and cancer healthcare system.

### Nurse outcomes

The primary outcome of this study is frequency of use of the evidence-informed symptom protocols. It is anticipated that they will be used for 75% of remote support for symptoms. Protocol use will be measured via chart audit. Six months after implementation, there will be an assessment of nurses’ satisfaction with their provision of symptom support using these new symptom protocols and their perception of the impact the protocols have had on their practice, including dialogue with patients. In addition to use of the protocol, completeness of the protocol and consistency of actions taken with evidence-based information in the protocol (*e.g.*, adherence to the guideline) in the chart audit will be measured.

### Patient outcomes

Patient outcomes that include confidence with managing their symptoms, symptom control, and satisfaction with remote support will be measured. Confidence with initiating self-care strategies will be assessed during the chart audit by determining the level of confidence documented on the symptom protocol (rated from 0 = no confidence to 10 = extremely confident). Symptom control will be measured by calculating the change in ESAS symptom severity score (from 0 to 10) from the initial contact to the follow-up contact a week later [10]. Patient satisfaction with remote support will be assessed using a valid and reliable instrument in the follow-up call at one week (Cronbach’s alpha coefficient = 0.64–0.93 for six subscales) [41].

### Healthcare-system outcomes

Through the chart audit being conducted as part of this study, the plan is to measure the impact on the healthcare system by determining the proportion of remote support contacts requiring higher level of care (*e.g.*, referral to the physician, in-person visits, emergency room visits, or hospitalizations) and the appropriateness of the health services used. This will allow for a comparison of health-system outcomes in patients for which a symptom protocol was used and those for which it was not used.

### Discussion of anticipated findings

We anticipate that our study will generate findings of interest to nurses, cancer programs, funders, cancer nursing organizations, and patients nationally and internationally. This will be the first known study to evaluate the implementation of symptom protocols for remote support in a cancer program and the first known study to determine whether these protocols are being used in a manner for which they were intended. Our study will enhance knowledge of common barriers and facilitators that are experienced by nurses when implementing symptom protocols for remote support, produce an implementation toolkit based on the effective intervention strategies used within three settings to overcome known barriers and facilitate protocol use, and establish a set of measures for ongoing monitoring of protocol use and outcomes at the level of the patient, nurse, and healthcare system. Furthermore, we plan to identify best practices to exemplify effective use of symptom protocols for remote support. Given that data will be based on three settings within three Canadian provinces, we anticipate that our findings will be transferable to others working in cancer programs that provide or are thinking about providing remote support.

## Competing interests

The authors declare that they have no competing interests.

## Authors’ contributions

DS (principal investigator) led the application for funding, together with other researchers (DB, MH, DH, CK, BS, AS) and knowledge users (MS, EG, AW, KC, BB, TM, JC). All authors contributed to study conception and participated in critically appraising and revising the intellectual content of the manuscript. All authors read and approved the final manuscript.

## Supplementary Material

Additional file 1 Figure S1.Example of one evidence-informed protocol for managing symptoms during cancer treatment. Protocols were developed using a rigorous methodology, informed by quality appraised clinical practice guidelines, and peer reviewed by nurses across Canada. Click here for file
