# Supplementary material for: Managing symptoms during cancer treatments: evaluating the implementation of evidence-informed remote support protocols
Source: Implement Sci. 2012 Nov 19;7:110. doi: 10.1186/1748-5908-7-110 (PMC3527220; doi:10.1186/1748-5908-7-110)
Supplement: Additional file 1 — Figure S1.Example of one evidence-informed protocol for managing symptoms during cancer treatment. Protocols were developed using a rigorous methodology, informed by quality appraised clinical practice guidelines, and peer reviewed by nurses across Canada. [file 1748-5908-7-110-S1.doc]

| **Nausea & Vomiting Protocol**  **Remote Assessment, Triage, and Management of**  **Nausea & Vomiting in Adults Undergoing Cancer Treatment**  (not for patients undergoing bone marrow transplant)  Nausea: A subjective perception that emesis may occur. Feeling of queasiness.  Vomiting: A forceful expulsion of stomach contents through the mouth and may include retching (gastric and esophageal movement without vomiting – dry heaves).^1,6^ | Name  Date of Birth  Sex  Hospital card number  Date and Time |
| --- | --- |

**1. Assess severity of nausea/vomiting (Supporting evidence: 3 guidelines)**

Tell me what number from 0 to 10 best describes your nausea

No nausea	0	1	2	3	4	5	6	7	8	9	10	Worst possible nausea ^8 (ESAS)^

Tell me what number from 0 to 10 best describes your vomiting?

No vomiting	0	1	2	3	4	5	6	7	8	9	10	Worst possible vomiting ^8 (ESAS)^

How worried are you about your nausea/vomiting?

Not worried	0	1	2	3	4	5	6	7	8	9	10	Extremely worried

**Ask patient to indicate which of the following are present or absent**

| Patient rating for nausea (see ESAS above)^8^ | 0-3 | 🞏 | 4-6 | 🞏 | 7-10 | 🞏 |
| --- | --- | --- | --- | --- | --- | --- |
| Patient rating for vomiting (see ESAS above)^8^ | 0-3 | 🞏 | 4-6 | 🞏 | 7-10 | 🞏 |
| Patient rating of worry about nausea/vomiting (see above) | 0-5 | 🞏 | 6-10 | 🞏 |  |  |
| How many times per day are you vomiting or retching?^1,7^  🞏No vomiting | ≤ 1 | 🞏 | 2-5 | 🞏 | >5 | 🞏 |
| Have you been able to eat within last 24 hours?^6,7^ | Yes | 🞏 | Yes, reduced | 🞏 | No | 🞏 |
| Have you been able to tolerate drinking fluids?^7^ | Yes | 🞏 |  |  | No | 🞏 |
| Are you feeling dehydrated, which can include feeling dizzy, a dry mouth, increased thirst, fainting, rapid heart rate, decreased amount of urine?^2,6^ | No | 🞏 |  |  | Yes | 🞏 |
| Do you have any blood in your vomit or does it look like coffee grounds?^6^ 🞏No vomiting | No | 🞏 |  |  | Yes | 🞏 |
| Do you have any abdominal pain?^6^ | No/Mild  0-3 | 🞏 | Moderate  4-6 | 🞏 | Severe  7-10 | 🞏 |
| Does your nausea/vomiting interfere with your daily activities at home and/or at work? Describe. | No | 🞏 | Yes, some | 🞏 | Yes, significantly | 🞏 |
|  | **Mild**  1 | | **Moderate**  2 | | **Severe**  3 | |
| **2. Triage patient for symptom management based on highest severity (Supporting evidence: 1 guideline)** ^6,7^ | 🞏 Review self-care. Verify medication use, if appropriate. | | 🞏 Review self-care. Verify medication use, if appropriate. Advised to call back if symptom worsens, new symptoms occur, or no improvement in 12-24 hours. | | 🞏 If 1 or more symptoms present with any vomiting or severe nausea, seek medical attention immediately. | |

If patient is experiencing other symptoms, did you also refer to the appropriate protocols? If yes, please specify:

**Additional Comments:**

Patient Name_______________

**3. Review medications patient is using for nausea/vomiting, including prescribed, over the counter, and/or herbal supplements (Supporting evidence: 5 guidelines)**

| Current use | Medications for nausea/vomiting | Notes (eg. dose, suggest to use as prescribed) | Type of Evidence |
| --- | --- | --- | --- |
| 🞏 | ondansetron (Zofran^®^), granisetron (Kytril^®^), dolasetron (Anszemet^®^)^1-5^ |  | Systematic review |
| 🞏 | metoclopramide (Maxeran^®^)^1,2,4,5^ |  | Systematic review |
| 🞏 | prochlorperazine (Stemetil^®^)^1,2,3,5^ |  | Systematic review |
| 🞏 | aprepitant (Emend^®^)^1-5^ |  | Systematic review |

**4. Review self-management strategies (Supporting evidence: 4 guidelines)**

| What strategies are already being used? | Strategy suggested/ education provided | Patient agreed to try | **Self-care strategies** |
| --- | --- | --- | --- |
| 1. 🞏 | 🞏 | 🞏 | What helps when you have nausea/vomiting? Reinforce as appropriate.  Specify: |
| 2. 🞏 | 🞏 | 🞏 | Are you taking sips of clear fluids (e.g. water, sports drinks, broth)?^6^ |
| 3. 🞏 | 🞏 | 🞏 | Have you tried relaxation techniques that may include guided imagery, music therapy, progressive muscle relaxation?^2,5,6^ |
| 4. 🞏 | 🞏 | 🞏 | Are you taking anti-emetic medications prior to your meals so that they are effective during and after meals?^5,6^ |
| 5. 🞏 | 🞏 | 🞏 | Are you trying to:  - eat 5-6 small meals?^5,6^  - eat foods that minimize your nausea and are your “comfort foods”?^5^  - avoid greasy/fried, highly salty, and spicy foods?^5,6^  - eat foods that are cold, avoiding extreme temperatures?^5,6^  - reduce food aromas and avoid other strong odors?^5,6^ |
| 6. 🞏 | 🞏 | 🞏 | Have you tried acupuncture or acupressure to help with your nausea/vomiting?^2,4,5^ (supporting evidence: systematic review) |
| 7. 🞏 | 🞏 | 🞏 | Would more information about your symptoms help you to manage them better? If yes, provide appropriate information or suggest resources. |

**5. Summarize and document plan agreed upon with caller (check all that apply)**

| 🞏 | No change, continue with self-care strategies and if appropriate, medication use |
| --- | --- |
| 🞏 | Patient agrees to try self-care items #:  How confident are you that you can try what you agreed to do (0=not confident, 10=very confident)? |
| 🞏 | Patient agrees to use medication to be consistent with prescribed regimen. Specify: |
| 🞏 | Referral (service & date): |
| 🞏 | Patient agrees to seek medical attention; specify time frame: |
| 🞏 | Advised to call back in 12-24 hours if no improvement, symptom worsens, or new symptoms occur |

| **Name** | **Signature** | **Date** |
| --- | --- | --- |

References

1. Kris, M., Hesketh, P., Somerfield, M., Feyer, P., Clark-Snow, R., et al. (2006). American Society of Clinical Oncology guideline for antiemetics in oncology: update 2006. *Journal of Clinical Oncology, 24*(18), 2932-2947. (AGREE Rigor score 85%) (updated 2011)
2. National Comprehensive Cancer Network. (2009). NCCN Clinical practice guidelines in oncology: antiemesis. Version 3. Retrieved from: http://www.nccn.org/professionals/physician_gls/f_guidelines.asp. (AGREE Rigor core 83%)
3. Antiemetic Subcommittee of the Multinational Association of Supportive Care in Cancer (MASCC). (2006). Prevention of chemotherapy- and radiotherapy-induced emesis: results of the 2004 Perugia International Antiemetic Consensus Conference. *Annals of Oncology, 17*(1), 20-28. (AGREE Rigor score 75%) (updated 2011)
4. Naiem, A., Dy, S., Lorenz, K., Sanati, H., Walling, A., & Asch, S. (2008). Evidence-based recommendations for cancer nausea and vomiting. *Journal of Clinical Oncology, 26*(23), 3903-3910. (AGREE Rigor score 68%)
5. Tipton, J., McDaniel, R., Barbour, L., Johnston, M., Kayne, M., et al. (2007). Putting evidence into practice: evidence-based interventions to prevent, manage, and treat chemotherapy-induced nausea and vomiting. *Clinical Journal of Oncology Nursing, 11*(1), 69-78. (AGREE Rigor score 57%)
6. Cancer Care Ontario. (2004). Telephone nursing practice and symptom management guidelines. Retrieved from: http://www.ontla.on.ca/library/repository/mon/9000/245778.pdf. (AGREE Rigor score 11%)
7. National Institutes of Health: National Cancer Institute. (2010). Common terminology criteria for adverse events (CTCAE) v4.03. Retrieved from: http://evs.nci.nih.gov/ftp1/CTCAE/About.html.
8. Bruera E, Kuehn N, Miller MJ, Selmser P, Macmillan K. The Edmonton symptom assessment system (ESAS): a simple method for the assessment of palliative care patients. J. Palliat Care 1991; 7(2):6-9.
